# Integrating Structure Propagation Uncertainties in the Optimization of Online Adaptive Proton Therapy Plans

**DOI:** 10.3390/cancers14163926

**Published:** 2022-08-14

**Authors:** Lena Nenoff, Gregory Buti, Mislav Bobić, Arthur Lalonde, Konrad P. Nesteruk, Brian Winey, Gregory Charles Sharp, Atchar Sudhyadhom, Harald Paganetti

**Affiliations:** 1 Harvard Medical School, Boston, MA 02115, USA; 2 Department of Radiation Oncology, Physics Division, Massachusetts General Hospital, Boston, MA 02114, USA; 3 Center of Molecular Imaging, Radiotherapy and Oncology (MIRO), Institute of Experimental and Clinical Research (IREC), Université Catholique de Louvain, 1200 Brussels, Belgium; 4 Department of Physics, ETH Zurich, 8092 Zurich, Switzerland; 5 Department of Radiation Oncology, Brigham and Women’s Hospital, Boston, MA 02115, USA

**Keywords:** proton therapy, online adaptation, deformable image registration, structure propagation

## Abstract

**Simple Summary:**

The fast and accurate definition of structures is a main limiting factor in online adaptive proton therapy. In this study, different methods to include structure propagation uncertainties in the optimization were compared with adaptation using physician-drawn structures, uncorrected propagated structures, and no adaptation. While adaptation with physician-drawn structures resulted in the best adaptive plan quality and no adaptation in the worst, manual structure correction could be replaced by a fast ‘plausibility check’, and plans could be adapted with correction-free adaptation strategies.

**Abstract:**

Currently, adaptive strategies require time- and resource-intensive manual structure corrections. This study compares different strategies: optimization without manual structure correction, adaptation with physician-drawn structures, and no adaptation. Strategies were compared for 16 patients with pancreas, liver, and head and neck (HN) cancer with 1–5 repeated images during treatment: ‘reference adaptation’, with structures drawn by a physician; ‘single-DIR adaptation’, using a single set of deformably propagated structures; ‘multi-DIR adaptation’, using robust planning with multiple deformed structure sets; ‘conservative adaptation’, using the intersection and union of all deformed structures; ‘probabilistic adaptation’, using the probability of a voxel belonging to the structure in the optimization weight; and ‘no adaptation’. Plans were evaluated using reference structures and compared using a scoring system. The reference adaptation with physician-drawn structures performed best, and no adaptation performed the worst. For pancreas and liver patients, adaptation with a single DIR improved the plan quality over no adaptation. For HN patients, integrating structure uncertainties brought an additional benefit. If resources for manual structure corrections would prevent online adaptation, manual correction could be replaced by a fast ‘plausibility check’, and plans could be adapted with correction-free adaptation strategies. Including structure uncertainties in the optimization has the potential to make online adaptation more automatable.

## 1. Introduction

Online treatment plan adaptation can improve dose conformity during the course of radiotherapy [1,2]. Treatment machines, such as MRI cobalt, MRI linacs [3,4], or Varian’s ETHOS [5], are specifically designed to enable online adaptive treatments. Online adaptation is most commonly applied to fast-changing or moving anatomies, such as pancreas, liver, lung, small nodes in the abdomen, head and neck (HN), or prostate cancer [6,7,8]. Early clinical trials show promising results in combination with hypofractionation and dose escalation, for example, for pancreas cancer patients [9].

Combining online adaptation with the superior low-dose characteristics of proton therapy is attractive, especially in anatomical areas that change quickly and are surrounded by multiple organs at risk, such as in the abdomen or head and neck regions. As a result, online adaptive proton therapy is being investigated by many research groups [10,11,12,13,14]. In the first experimental validation of online adaptive proton therapy [15], the application was limited to the head to avoid deforming structures and manual contour corrections. Other proposed online adaptive proton therapy workflows include structure deformations using deformable image registration (DIR) [16]. However, the uncertainty introduced by using DIR for structure propagation or dose restoration has not been well-quantified, and the impact on dose adaptation has not been studied.

In current clinical workflows, online adaptation requires manual structure approval, which is often resource- and time-consuming. DIR [17] or auto segmentation [18] can be used to generate structures on daily images, but the structures generated by these algorithms must be manually reviewed and corrected before plan optimization. This step takes about 5 to 10 min [19] and is currently one of the main bottlenecks for the clinical implementation of online adaptive therapy.

This study investigates whether uncertainties from propagating structures with DIR can be integrated directly into the optimization of adaptive plans. Different automatable adaptive strategies were compared to avoid manual contour correction for online adaptive proton therapy that combine the information of multiple DIR algorithms. The proposed strategies were compared with a simplified adaptation strategy that uses uncorrected deformed structures, adaptive plans optimized with manually defined and corrected structures, as well as non-adapted plans.

## 2. Materials and Methods

### 2.1. Patient Data and Treatment Planning

The different adaptation strategies were tested on a cohort of 16 patients with 3 different disease sites. All patients included in this study received some form of adapted radiotherapy. All images used for adaptation for these patients were also used in this study. The CTV volumes cover a wide range and are given in Table 1.

**Figure 1:**
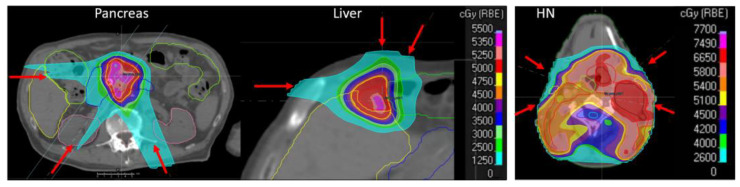
Example treatment plans for pancreas, liver, and HN patients. The red arrows depict the beam directions.

Six pancreatic cancer and five liver cancer patients, previously treated in breath hold with online adaptive photon therapy at an MRI linac, were replanned for multi-field optimized intensity-modulated proton therapy (IMPT) plans in Raystation version 8.99 (Raysearch, Stockholm, Sweden). Similar to the original MR linac treatment, the original treatment plans were calculated on deformed planning CTs, which were registered to the planning MRI using MIM (MIM Software Inc., Cleveland, OH, USA). The daily replanning CTs for adaptation were generated by applying the ViewRay (ViewRay, Oakwood Village, OH, USA) DIR vectors from the registration of the daily MRI to the planning MRI, to the planning CT that was deformed to match the planning MRI. Air and soft tissue that differed on the deformed CT with respect to the planning MRI were overwritten, with mass densities of 0.0012 g/cc and 1.02 g/cc, respectively. Areas containing density overwrites were avoided in the IMPT beam angle selection. For the proton plans, the original stereotactic prescription (50Gy-RBE in 5 fractions; RBE = 1.1) and the original organ constraints were used (Table 2). The treatment plans consisted of 3 beams (Figure 1, left).

Furthermore, treatment plans for 5 head and neck (HN) cancer patients, previously treated by adaptive standard fractionated VMAT, were optimized for IMPT using the original planning CT image. Each HN patient had one repeated CT during the course of treatment. The dose prescription of 70 Gy-RBE to the high-risk clinical target volume (CTV) and 54 Gy-RBE in 30 fractions to the low-risk CTV was optimized with 4 beams (Figure 1, right). All proton plans were optimized with robust optimization on the CTV, with a 3% range uncertainty and 3 mm setup uncertainty. The organ constraints are given in Table 2.

### 2.2. Structure Propagation

Three DIR algorithms were included in this study: Raystation Anaconda and two open-source registration algorithms from Plastimatch (www.plastimatch.org (accessed on 1 June 2022)), one using B-spline and one using Demons registration. For the structure propagation of pancreas and liver patients, the 5 daily MRI images were registered to the planning MRI with each DIR algorithm. The inverse registration vector was used to propagate the structures. The propagated contours were copied rigidly to the corresponding replanning CTs from the original MR linac treatment.

For HN patients, a single additional CT was available for one-time replanning during the course of treatment. This repeated CT was first rigidly pre-registered and then registered with all three DIR algorithms to the planning CT. Structures were propagated from the planning to the replanning CT using the inverse registration vectors. The adaptive plans were optimized directly on the rigidly registered replanning CT.

### 2.3. Adaptive Strategies

**Figure 2:**
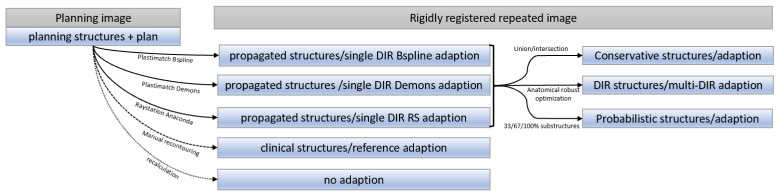
Scheme of the workflow. Doses were calculated on rigidly pre-registered repeated images. Three different DIRs were applied for structure propagation between this repeated image and the planning images. The structures propagated with these DIRs were used for ‘single-DIR plan adaptation’. Information from all 3 DIRs was combined by using their union (for organs and HN target) and intersection (liver and pancreas target) structures for the conservative plan adaptation approach, and substructures were defined according to the frequency that each voxel was defined as belonging to a certain structure for the probabilistic optimization. All adaptive plans were evaluated using the clinical structures drawn by a physician.

The propagated structures with different DIR algorithms show differences between each other and the clinical contours. As manual structure corrections require a lot of resources and time, it is desirable to integrate the potential DIR-related structure propagation uncertainties directly into the optimization of the adaptive plan. To make plans more robust against DIR-induced structure uncertainties, different strategies to mitigate contour propagation uncertainties were compared. The beam angles, planning constraints, and weights were identical to those used for the initial proton treatment plan. A schematic of how the different structures were generated and used for optimization is given in Figure 2.

- Single-DIR adaptation:
  DIR-propagated structures were directly used for optimization without any manual corrections. Therefore, 3 different single-DIR adaptive plans were optimized on all replanning CTs, with structures deformed with Raystation, Plastimatch Demons, and Plastimatch B-spline.

- Multi-DIR adaptation:
  The same replanning CTs with the 3 different structure sets used for single-DIR adaptation described above were combined using the Raystation robust optimization function on multiple images and structure sets. This is the worst-case optimization [20], optimizing the plan using multiple images and structure sets in parallel.

- Conservative adaptation:
  Structures from the 3 different DIRs were combined. For pancreas and liver cancer patients, a stereotactic prescription was used, i.e., all organ constraints must be fulfilled, while target coverage is only the second priority. Therefore, for the conservative adaptation of these stereotactic prescriptions, the intersection of all propagated structures was used as the target structure, and the union for organs. In contrast, for HN patients with this prescription, the target coverage had a higher priority; therefore, the union of all propagated structures was used for the target and organs. The union and intersection of structures were calculated in Plastimatch and imported into Raystation for optimization.

- Probabilistic adaptation:
  Substructures were defined for each structure depending on how often a voxel was classified as part of the structure. If all 3 DIRs agreed that a voxel was a target, this voxel was included in the 100%-target substructure; if only two DIRs classified a voxel as a target, it belonged to the 67%-target substructure; if only one algorithm defined it as a target, it belonged to the 33%-target substructure. The 100%, 67%, and 33% substructures were calculated in Plastimatch and imported into Raystation. The optimization constraints of each structure were identical to those used for the planning CT, but the weights of these substructures varied according to the frequency that voxels were classified as a target or organ amongst the DIRs [21].

- Reference adaptation:
  All replanning CTs had “clinical” structures, manually contoured by a physician. These structures were directly used for the optimization of reference adaptive plans. These reference adaptive plans should result in the best possible treatment plan.

- No adaptation:
  To compare the effect to that of a non-adaptive approach, which can be seen as the worst case, treatment plans were recalculated on the replanning CTs without plan re-optimization.

All plans were optimized and calculated in Raystation using different optimization structures, and all evaluations were performed using the clinical structures. If voxels were assigned to multiple structures, they were kept in both and handled by the optimizer in the objective function without supervision. Indeed, one of the aims of this study was to compare the handling of uncertain voxel assignments directly by the different optimizations (e.g., probabilistic optimization or multiple DIR optimization) compared with manual voxel assignment (e.g., by a physician or one DIR algorithm).

### 2.4. Plan Quality Scoring

To simplify and standardize the comparison of multiple plans, the following scoring system was used: if the prescribed constraint $C_{ref}\;$ (listed in Table 2) was not fulfilled, the difference of constraint C to reach the prescription $C_{ref}\;$ was summed: $score=C1-C1_{ref}+C2-C2_{ref}+\ldots +Cn-Cn_{ref}$.

Similar to an objective function during plan optimization, this scoring system adds absolute values with different units. Therefore, the resulting score has arbitrary units (au) and depends on how many constraints are applied, specific to the anatomical site. For example, if, for HN patients, the dose to the brainstem exceeded 54 Gy by 1 Gy, it would contribute ‘1’ to the score; if the high-risk CTV V74.9 exceeded 1cc by 0.1cc, that would contribute ‘0.1’ to the score. As HN patients have more constraints than liver or pancreas cancer patients, the calculated scores were higher. Scores were calculated for each plan using the clinical structures. To reduce the patient dependency (e.g., some patients having larger tumors that were closer to organs than others), the difference between the score and the reference score of the plan adapted with clinical structures was calculated.

The scoring only provided a relative ranking between different adaptive plans. The mean and range of all scores for all patients and fractions with the same adaptive strategy were reported. The lower the score, the better clinical constraints and prescriptions were fulfilled.

## 3. Results

**Figure 3:**
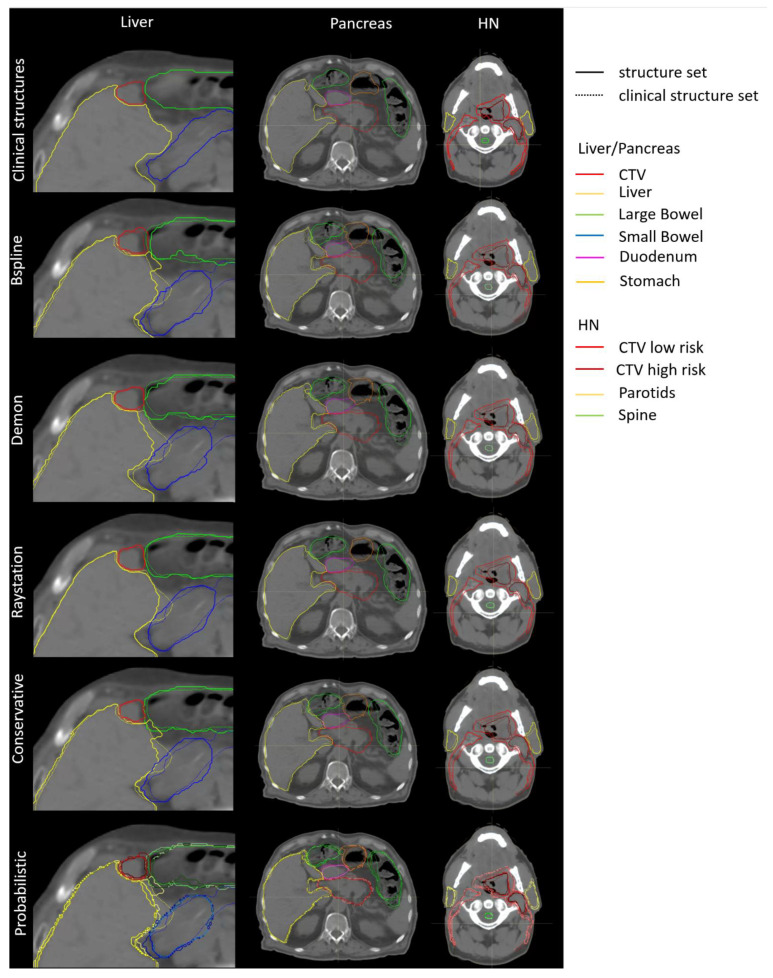
Example of the clinical, deformed, conservative, and probabilistic structures set for each indication. The clinical reference is overlayed in dashed lines with the propagated structures.

The different deformed structures generally had a good agreement with the clinical structures. However, some voxels were defined as part of a certain structure by the physician, but not by any of the deformed structures. Therefore, there was a risk that, even when multiple DIR algorithms were applied, the optimization may not have included all voxels encompassed by physician-drawn structures, potentially risking target underdosage or organ overdosage. This is especially relevant for maximum or minimum constraints, as already small structural differences can lead to higher maximum doses in certain organs or leave parts of the target uncovered. Examples of the propagated structures are given in Figure 3.

**Figure 4:**
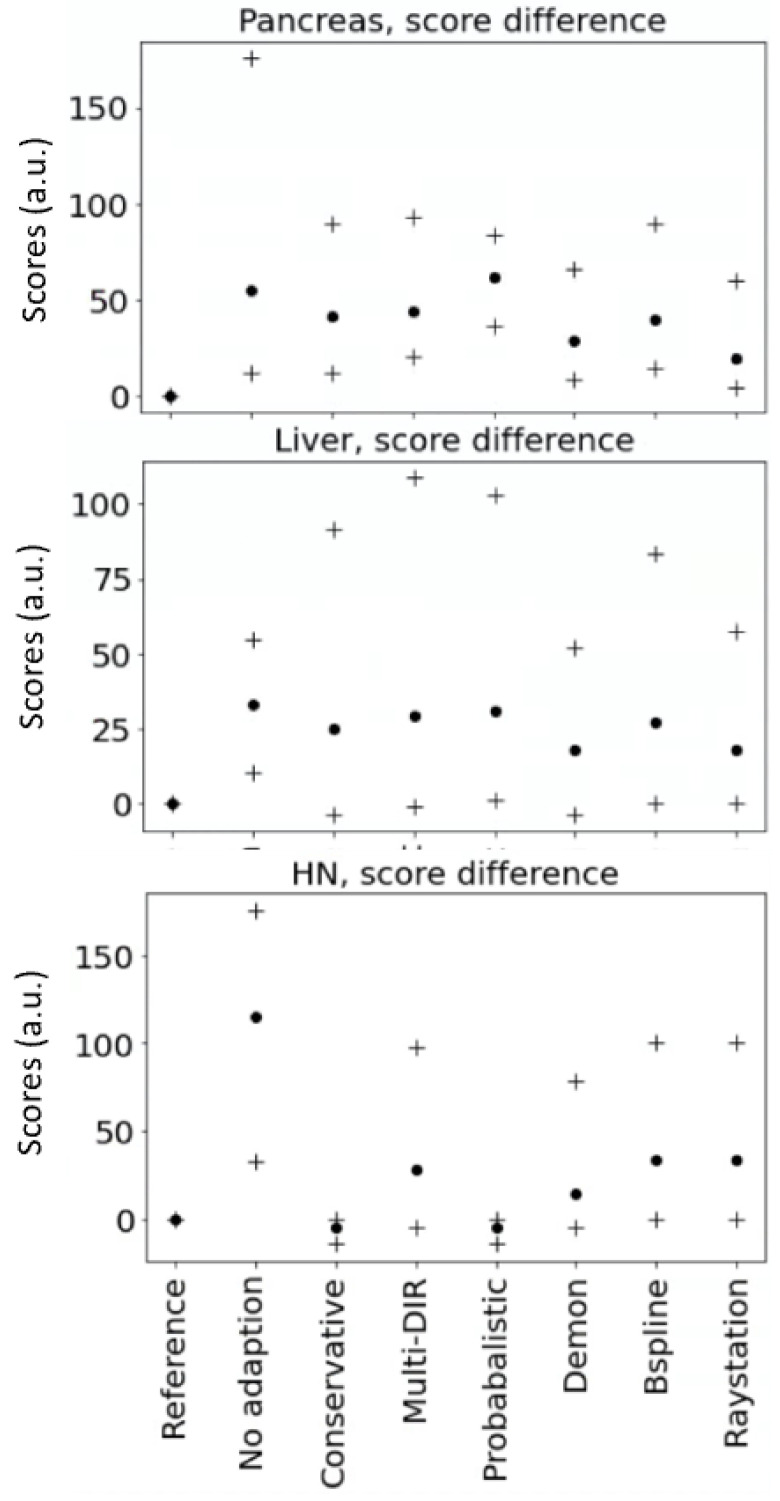
Minimum, maximum (crosses), and mean (dots) of the score differences from the reference plan score optimized on clinical structures. Score units are arbitrary and depend on the tumor location and the number of constraints.

Figure 4 shows how the adaptive strategies performed compared with the reference adaptive plan optimized with clinical structures. For each adaptive strategy, the score difference with the reference adaptation plan is reported, averaged over all patients and adaptive images with the same indication. The scores differed between the different locations depending on the tumor position, e.g., the liver tumors were either close to the duodenum or the large bowel, the pancreas was surrounded by multiple gastro-intestinal (GI) organs, and HN tumor treatments involved more constraints and dose levels and, therefore, higher scores.

Overall, plan adaptation improves the dose distribution compared with no adaptation. Adaptation with the clinical structures, which were also used for evaluation and scoring, performed best. No adaptation generally resulted in the worst scores, especially for HN patients. The average score difference without adaptation to the reference adaptation was 54 au for pancreas, 33 au for liver, and 115 au for HN. Adaptation with a single DIR improved the scores compared with no adaptation (pancreas 19–39 au/liver 17–27 au/HN 15–34 au). The best performing algorithm was patient-dependent. The probabilistic and conservative adaptations were very effective for HN patients, as both strategies showed a score difference down to 5 au, but not for pancreas and liver patients. This was due to the stereotactic prescription, where organ constraint optimization weights were higher than the target constraint weights.

## 4. Discussion

In this study, different strategies to include structure uncertainty in the adaptive plan optimization (three single-DIR adaptations, multi-DIR adaption, conservative, probabilistic, adaptation with clinical structures, and no adaptation) were implemented and evaluated for a cohort of pancreas, liver, and HN cancer patients. In principle, online optimization based on physician-corrected or manually redrawn structures is the most accurate solution. However, if the required time and personnel resources for contour correction would prevent online adaptation in clinical practice, automatic structure QA and correction-free optimization can be valuable alternatives to manual structure definition and correction. For the HN cancer patients, including the structure propagation uncertainties with the probabilistic or conservative strategies improved the adaptation quality over a single-DIR adaptation. For the abdominal indications, we observed no additional benefit of the probabilistic or conservative strategy over single-DIR adaptation. However, with the good image contrast of these abdominal MRI images and HN CTs, adaptation on uncorrected propagated structures improved plan quality compared with no adaptation at all. The acceptable results of optimization on uncorrected deformed structures with a single-DIR algorithm agreed with previous studies, for example, those by Elmahy et al. and Qiao et al. [22,23] for prostate cancer, or that by Nenoff et al. [24] for lung cancer.

In this study, the propagated structures were all reasonable and did not show extreme deviations from the clinical structures. To avoid outliers, a short visual check and/or automatic ‘plausibility checks’, for example those proposed in [15], are recommended for all structures.

Online adaptive therapy improves the treatment conformity and enables dose escalation for pancreatic cancer patients [9,25], given that, in the abdomen, the anatomy changes daily and the GI organs can have large position differences relative to the tumor. However, DIR algorithms can also have considerable uncertainties [17]. For the GI patients investigated here, all five fractions were delivered within 1 week. The large weight losses and ascites that often occur in abdominal cancer patients did not take effect in such a short time frame; therefore, the effect on the non-adapted plan was limited.

In contrast, for the investigated HN patients, the adaptive replanning CTs were acquired multiple weeks after the planning image. The patients showed weight loss, resulting in changes in the outer contours. All DIR algorithms showed a good agreement with the manually corrected structures, since this anatomical area is dominated by bony structures and the shrinkage of the external contour. Therefore, the effect of adaptation was also larger than that for the investigated abdominal patients.

In this study, structural uncertainties were considered representative of the inter-algorithm variability between different DIR algorithms. The DIR algorithms used in this study used different optimization methods, but they were all based on image intensity and had smoothing parameters to restrict the differences between neighboring voxels (sliding surfaces); therefore, they might be systematically closer to each other than to clinical structures. The use of a biomechanical algorithm, such as Raystation Morpheus [26], was not considered, given that it requires structure sets to be defined on both registration images, which requires a similar effort to manual correction and is not suitable for structure propagation.

The proposed strategies of including structure propagation uncertainty in the plan optimization could also be applied to other structure uncertainty definitions, not only adaptive planning, but also initial planning. For example, differences between multiple auto segmentation contours or inter-observer variabilities in contouring [27] could be included in the optimization.

The probabilistic optimization showed very good results for HN patients, but not for the abdominal indications. The poor performance in the abdominal patients might be due to the stereotactic prescription, where the weights of the organ constraints were more important than the constraints to ensure target coverage. The stereotactic prescription was also the reason for the different definitions of the target in the conservative planning approach. In stereotactic GI treatments, organ constraints are the most important, even if they come at the cost of target coverage. Therefore, conservative adaptive plans for the pancreas and liver were optimized on the smallest possible target with the largest possible organs. For HN patients, target coverage has higher importance, and the largest possible target was used in the conservative optimization.

The structural changes in the pancreas and liver patients are mostly caused by interfractional bowel and GI movement, combined with some residual daily breath hold variations. Due to the prescription of five times 10 Gy, the treatment takes place within one week; therefore, the tumor and OAR volumes mostly shift around, but do not change volumes. In contrast, the HN adaptive images were acquired some weeks after the planning image, and the patients showed relevant weight loss. Because of these larger changes, the non-adaptive plans for HN patients show a severely decreased plan quality, resulting in a large benefit of adaptation.

For liver cancer patients, the adaptive scenarios showed slightly better average scores, but worse maximum scores, than no adaptation. The maximum points all resulted from one fraction of one patient. For this fraction, there were some voxels of the target defined as ‘stomach’ by all three DIR algorithms that were, therefore, spared in the adaptive scenarios. In this fraction, the non-adapted plan had better target coverage and, despite the increased stomach dose, resulted in better scores. This case shows one of the limitations of the proposed scoring system.

The plans were compared using a scoring system, which simplified the comparison of multiple plans with different planning constraints. It is understood that a single scoring number can never provide the full picture of a complex plan comparison. However, it is a useful tool to standardize the comparison of multiple plans and rank them relative to each other. The calculation of the scores is, therefore, crucial for interpreting the results. In this case, a linear scoring system was used with equal scoring weights between all clinically used constraints. To test the stability of the results against the influence of individual constraints, the scoring weights of the individual constraints were modified. The weight of the constraint varied between all being weighted equally (as used in this study) and the target or organs being weighted up to 10 times more relative to other structures. The resulting score variations were small and did not change the conclusion of this study.

## 5. Conclusions

We investigated different strategies to include structure uncertainties in adaptive plan optimization. Adaptation with the clinical structures used for evaluation resulted in the best adaptive plan quality. If the time required for manual correction would prevent or degrade online adaptation in clinical practice, it could be replaced by a fast planning approach using correction-free adaptation. Adaptation on propagated, uncorrected structures showed a benefit over no adaptation. Including structure propagation uncertainties in the optimization further improved the optimized plan over optimizing with single-DIR propagated structures for the investigated HN patients. This has the potential to overcome manual structure correction and make online adaptation more automatable, reduce the required resources, and, therefore, make it more widely accessible.

## Figures and Tables

**Table 1:** Average (minimum and maximum) CTV volumes in cm^3^ of all patients by indication.

Pancreas CTV	Liver CTV	HN High-Risk CTV	HN Low-Risk CTV
63.3 (14.7–125.1)	113.6 (20.7–340.1)	105.1 (61.2–192.3)	353.7 (243.3–469.2)

CTV: clinical target volume; HN: head and neck.

**Table 2:** Planning constraints for pancreas, liver, and HN cancer patients.

	Structure	Constraint	Importance
**Liver and pancreas**	CTV	V47.5Gy > 95%	Soft constraint
	Stomach	V33Gy < 1 cc	Hard constraint
	Small bowel	V33Gy < 1 cc	Hard constraint
	Large bowel	V33Gy < 1 cc	Hard constraint
	Duodenum	V33Gy < 1 cc	Hard constraint
	Spinal Cord	V25 < 0.5 cc	Hard constraint
	Kidneys	mean < 10Gy	Hard constraint
	Liver (-GTV)	mean < 20Gy	Hard constraint
		V_tot_-V15 > 700 cc	Hard constraint
**HN**	High-risk CTV	V66.5Gy > 95%	Hard constraint
		V74.9 < 1 cc	Hard constraint
	Low-risk CTV	V51.3 > 95%	Hard constraint
		V57.8 < 1 cc	Soft constraint
	Brainstem	max < 54Gy	Hard constraint
	Spinal cord	max < 45Gy	Hard constraint
	Constrictors	mean < 42Gy	Hard constraint
	Larynx	mean < 40Gy	Hard constraint
	Parotids	mean < 26Gy	Hard constraint

CTV: clinical target volume; HN: head and neck.

## Data Availability

The data are not publicly available due to privacy restrictions.
